# Automated detection of residual cells after sex-mismatched stem-cell transplantation – evidence for presence of disease-marker negative residual cells

**DOI:** 10.1186/1755-8166-2-12

**Published:** 2009-05-29

**Authors:** Jörn Erlecke, Isabell Hartmann, Martin Hoffmann, Torsten Kroll, Heike Starke, Anita Heller, Alexander Gloria, Herbert G Sayer, Tilman Johannes, Uwe Claussen, Thomas Liehr, Ivan F Loncarevic

**Affiliations:** 1 Jena University Hospital, Institute of Human Genetics and Anthropology, Kollegiengasse 10, D-07743 Jena, Germany; 2 Clondiag Chip Technologies, Loebstedter Str. 103–105, 07749 Jena, Germany; 3 Interdisciplinary Centre for Bioinformatics, University of Leipzig, Härtelstr. 16–18, 04107 Leipzig, Germany; 4 Clinic of Internal Medicine II, Oncology and Hematology, University Medical Centre Jena, Erlanger Allee101, 07747 Jena, Germany; 5 MetaSystems GmbH, Robert-Bosch-Str. 6, 68804 Altlussheim, Germany

## Abstract

**Background:**

A new chimerism analysis based on automated interphase fluorescence in situ hybridization (FISH) evaluation was established to detect residual cells after allogene sex-mismatched bone marrow or blood stem-cell transplantation.

Cells of 58 patients were characterized as disease-associated due to presence of a bcr/abl-gene-fusion or a trisomy 8 and/or a simultaneous hybridization of gonosome-specific centromeric probes. The automatic slide scanning platform Metafer with its module MetaCyte was used to analyse 3,000 cells per sample.

**Results:**

Overall 454 assays of 58 patients were analyzed. 13 of 58 patients showed residual recipient cells at one stage of more than 4% and 12 of 58 showed residual recipient cells less than 4%, respectively. As to be expected, patients of the latter group were associated with a higher survival rate (48 vs. 34 month). In only two of seven patients with disease-marker positive residual cells between 0.1–1.3% a relapse was observed. Besides, disease-marker negative residual cells were found in two patients without relapse at a rate of 2.8% and 3.3%, respectively.

**Conclusion:**

The definite origin and meaning of disease-marker negative residual cells is still unclear. Overall, with the presented automatic chimerism analysis of interphase FISH slides, a sensitive method for detection of disease-marker positive residual cells is on hand.

## Background

Malignant hematological diseases represent 5.5% of all cancers in Germany [1]. One way to cure these fatal diseases is allogenic bone marrow or blood stem cell transplantation. In case of a male donor and female recipient (and vice versa) we talk of a sex-mismatched transplantation. In such a setting it is relatively simple to classifiy donor and acceptor cells in the bone marrow or blood cell system. The existence of 100 percent donor cells is called complete chimerism, in contrast a mixture of both donor and acceptor cells mixed chimerism. Chimerism analysis is done on these sex-mismatched transplants to monitor minimal residual disease and to plan further immunotherapy like donor lymphocyte infusion (DLI) [2]. In routine diagnostics, fluorescence in situ hybridization (FISH) is frequently applied for chimerism analysis [3-6], which demands manual time-consuming counting of cells. An experienced technician needs about 2.5 hours for approximately 3,000 cells. Therefore, an automatic FISH chimerism analysis is extremely valuable for diagnostics and correct treatment of affected patients, as it can be carried out in a fraction of time. Thus, the presented single cell based approach becomes now competitive in comparison to PCR based chimerism analysis [7].

Frequently observed disease-markers are the bcr/abl-fusion-gene as present in more than 95% of chronic myeloid leukemia (CML) cases [8,9], and trisomy 8 found in 11% of acute myeloid leukemia (AML) [10]. The simultaneous detection of the gonosomal constitution and a tumor marker enables the identification of residual tumor cells. The latter was already proposed 1994 by Nagler and coworkers [11], however, it was not often carried out before [12-14], and not studied under routine conditions. Here we tested an automated interphase FISH analysis for the characterization of chimerism in 58 patients after allogenic stem cell transplantation with different hematological malignancies.

## Results

### Determination of cut off levels

FISH-analysis of residual cells after sex-mismatched transplantation is mainly based on simultaneous labeling of the centromeres of the X- and Y-chromosomes. Because of possible false positive and false negative results e.g. due to background or hybridization problems, it was necessary to determine the cut off level. Therefore, a total of 26,633 cells from 10 healthy female and 35,783 cells from 11 healthy male were analyzed with the described automated system. The automated analysis showed in the female controls 257 cells with apparent male signal constellation (XY), and the male controls had 142 cells with apparent female signal constellation (XX). To control these automated results we investigated all questionable cells; only 38 out of 257 and 27 out of 142 could be confirmed to be real XY-positive cells or in the male case XX-positive cells. This corresponds to a false positive rate of 0.14% in female and 0.08% in male. An additional random control of 4,841 XX cells in female and of 4,535 XY cells in male showed that there was no further failure of automatic counting.

**Figure 1:**
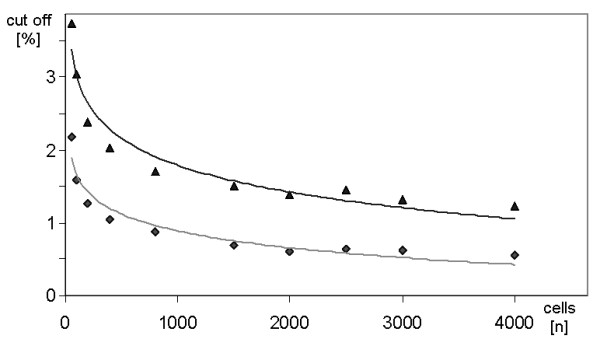
**Cut off level in dependence of the number of evaluated cells (n) in female (gray line, diamonds) and male (black line, triangles)**.

As the cut off level depends on the amount of analyzed cells, all mentioned female and male cells were listed in spreadsheets with random order and arranged in blocks of 50, 100, 200, 400, 800, 1,500, 2,000, 2,500, 3,000 and 4,000 cells. Subsequently, the mean and standard deviation was assessed for each block. The cut off level was defined as the mean plus twice the standard deviation. The respective cut of levels for each block size were fitted by a trend line enabling the calculation of cut off levels for arbitrary cell numbers up to 4000. Fig. 1 shows the determined/calculated cut off values for female and male cells including trend lines.

In order to determine the false positive rate for trisomy 8 another 15,882 cells from 5 healthy people were analyzed with centromere 8 probes. The mean false positive rate was 1.2%. In the same manner 11,453 cells of 11 healthy controls were investigated using the LSI-probe against the bcr/abl-fusion gene. The mean false positive rate for the bcr/abl-probe was 0.7%. For estimating the cut off level for XX/XY in combination with trisomy 8 (XX or XY+trisomy 8) or bcr/abl (XX or XY+bcr/abl) the 95-quantil with the following formula was used:



Thus, the cut off level was as follows:

• for XX+trisomy 8 and XX+bcr/abl = 0.005%

• for XY+trisomy 8 and XY+bcr/abl = 0.003%.

### Minimum number of cells to be analyzed in a blood sample

Two statistical aspects of the present study were further assessed. First, the minimum number of cells to be analyzed was determined in order to achieve a predefined accuracy for the estimated fraction of acceptor cells in the total blood of the patient.

Given the total blood of a transplanted patient consists of N cells of which N_A _are acceptor cells, the fraction of acceptor cells in the patient is P_A _= N_A_/N. A blood sample contains fewer than N cells and the fraction of acceptor cells in the sample p_A _can only be an estimate for the true fraction P_A_. But how many cells must be analyzed in a blood sample in order to achieve a predefined accuracy? The probability of finding M_A _acceptor cells in a blood sample of size M drawn from a total of N blood cells of which N_A _are acceptor cells is given by a hypergeometric distribution. However, the sample size M is generally much smaller than the total number of cells N. Thus, the true fraction of acceptor cells P_A _can be assumed to be the same before and after the blood sample has been drawn from the patient. The hypergeometric distribution is then well approximated by the binomial distribution . In the present case the exact value of P_A _= N_A_/N is unknown but is to be estimated by the sample ratio p_A _= M_A_/M. The mean and standard deviation of the random variable p_A _are given by , respectively. For determining the minimal number of cells to be analyzed in a blood sample we use the order of magnitude estimate P_A _= 0.01 according to the measurements. The coefficient of variation (equivalent to the relative standard deviation given in %) is then given by the ratio of standard deviation and mean . For the relative standard deviation to be smaller than q the sample size must exceed M* = 99/q^2^. Hence, for q = {100%, 50%, 25%} the required sample sizes must be larger then M* = {99, 396, 1584}, respectively.

### Error bounds for the fraction of acceptor cells due to classification errors of the automated cell recognition device

**Figure 2:**
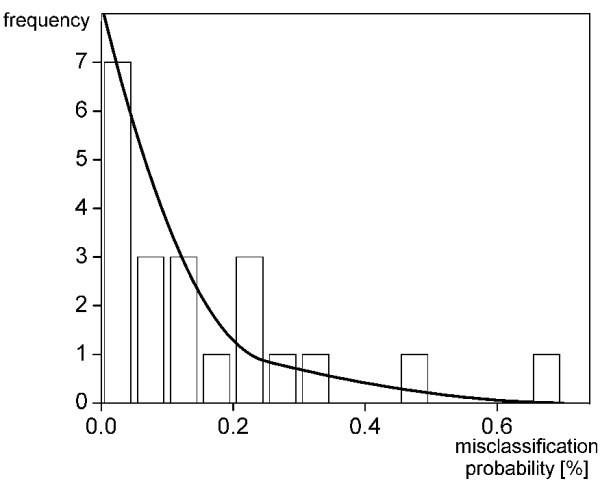
**Histogram for the misclassification error as estimated from the number of wrongly classified cells per sample**. Blood samples were obtained from 10 healthy females and 11 healthy males. The histogram is fitted by a sum of two beta distributions.

Second statistical aspect, the error bounds for the fraction of acceptor cells in the blood sample were calculated with respect to classification errors introduced by the automated cell recognition device. The measured number of acceptor cells may not reflect the real number of acceptor cells in the sample due to measurement errors of the automated device. In order to assess the error rate of the device we classified 10 and 11 samples from healthy females and males, respectively, and obtained histograms for the fraction of misclassified cells. These were similar for females and males and thus pooled in a single distribution as shown in Fig. 2. Using this error distribution we calculated the probability density function for the true number of acceptor cells in the sample as described in the following paragraph. The resulting probability density function indicates that the measurements rather overestimate the number of true acceptor states.

### Probability density function for the true number of acceptor cells in the sample

In order to assess the error introduced by the measuring device we calculated the probability distribution  for the true number of acceptor cells M_A _in the sample given the number of measured acceptor cells M*_A_. First note that for a given misclassification probability b the number of correctly measured acceptor cells M*_AA _is binomial distributed with total number of cells M_A _and probability 1-b. Accordingly, the number of donor cells erroneously measured as acceptor cells M*_AD _is binomial distributed with total number of cells M_D _and probability b. As shown in Fig. 2 the misclassification probability b itself is *β*-distributed. Thus, b-averaged binomial probability distributions are obtained by integration according to the *β*-distribution, i.e.



The probability of measuring M*_A _acceptor cells conditional on the fact that M_A _true acceptor cells are present in the sample is given by the sum of probabilities consistent with the conditions M*_A _= M*_AA _+ M*_AD _and M = M_A _+ M_D_. It reads



in which the product  is implied by the fact that the events 'M*_AA _out of M_A_' and 'M*_AD _(= M*_A _- M*_AA_) out of M_D _(= M - M_A_)' are independent and must occur at the same time. The probability for the true number of acceptor cells M_A _in the sample conditional on M*_A _acceptor cells having been measured is given according to Bayes' theorem



From the pooled experimental data the *a priory *probability  for the number of measured acceptor cells was estimated to be a sum of two exponentials. In the present medical context the prior for the number of acceptor cells in the sample *f*_*M*_(*M*_*A*_) is best chosen to be uninformative (i.e. constant) in order not to bias the results towards low M_A _values, which would conflict with the interest of the patient. Another choice would be to equate . Since  is strongly peaked for zero measured acceptor cells the resulting  is biased towards low M_A_-values, especially for small sample sizes M. This choice could be more interesting for e.g. insurance companies.

**Figure 3:**
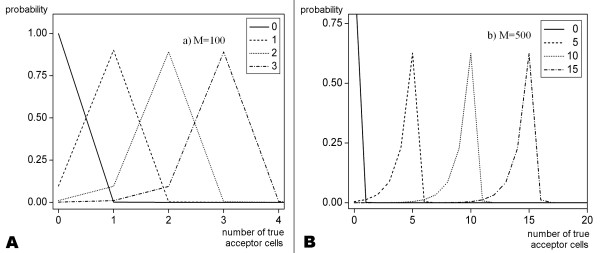
**Sample plots for the conditional probability distribution  for the number of true acceptor cells M_A _in the sample for sample sizes M = 100 (A) and 500 (B)**. The prior is uninformative. The different curves correspond to different measured values M*_A _as indicated in the legends. These values correspond to 0%, 1%, 2% and 3% of the corresponding sample size. The distributions are biased to lower values of M_A _(see text).

Sample plots of the conditional probability distribution  are shown in Fig. 3 for the empirical prior. From these plots it becomes clear that the number of acceptor cells is rather overestimated by the measurements since the great majority of cells are donor cells that are occasionally classified as acceptor cells. The opposite case, i.e. acceptor cells being classified as donor cells, is very rare simply because there are only very few acceptor cells.

### Quantification and characterization of residual cells in patients after sex-mismatched transplantation

Overall 454 samples of 58 patients were investigated with X- and Y-chromosome specific probes as described in detail in Tab. 1. 33 patients had a complete chimerism (posttransplant 290 samples) and therefore no residual cancer cells. 25 had a mixed chimerism (47 of 163 posttransplant samples). In order to see the clinical relevance of residual cells, patients with mixed chimerism were divided in two groups, one with less than 4% sexmismatch to the donor (13 patients) and the other with more than or equal 4% sexmismatch (12 patients). Both groups show no correlation with the time since transplantation. Patients with < 4% residual cells were transplanted between 10–55 years (median = 46), patients with ≥ 4% residual cells between 0–66 years (median = 49).

**Table 1:** Patient characteristics

**patient**	**sex**	**primary**	**Tx**	**conditioning**	**PBSCT/**	**death**	**reason**	**cytogenetic**	**number analysed**
**no**.		**disease**	**age**		**BMT**		**death/day**	**marker**	**probes**
**1**	m	SAA	10	n.k.	PBSCT	yes	n.k./+168		**1**
**2**	f	AML	62	M	PBSCT	no			6
**3**	f	CML	51	C	PBSCT	yes	GvHD/+575	bcr/abl	2
**4**	m	MDS	37	M	PBSCT	no			5
**5**	f	AML	44	M	PBSCT	no			3

**6**	f	ALL	43	C	BMT	no			5
**7**	f	NHL	39	M	PBSCT	no			19
**8**	f	CML	25	C	PBSCT	no			21
**9**	f	AML	49	C	PBSCT	no			3
**10**	f	MM	57	C	PBSCT	no			7

**11**	f	CML	12	n.k.	PBSCT	yes	n.k./+110		3
**12**	f	CML	43	n.k.	PBSCT	no			1
**13**	m	OP	0	n.k.	BMT	no			4
**14**	f	AML	41	M	PBSCT	no		trisomy 8	17
**15**	m	SAA	49	M	PBSCT	no			18

**16**	f	AML	55	M	PBSCT	yes	infection/+458		3
**17**	m	MM	40	M	BMT	yes	relapse/+278		8
**18**	f	AML	48	M	PBSCT	yes	relapse/+125		3
**19**	m	MM	60	M	PBSCT	yes	infection/+82		2
**20**	f	AML	25	M	PBSCT	no			15

**21**	m	ALL	34	C	PBSCT	yes	GvHD/+117		1
**22**	m	CML	39	C	PBSCT	no		bcr/abl	3
**23**	f	MDS	52	M	PBSCT	no			15
**24**	f	CML	1	n.k.	BMT	no			3
**25**	f	ALL	14	C	PBSCT	yes	n.k./+361		2

**26**	m	AML	89	M	PBSCT	no			4
**27**	f	AML	49	C	PBSCT	no			14
**28**	f	AML	48	M	PBSCT	yes	infection/+144		2
**29**	m	CML	49	C	PBSCT	yes	GvHD/+833	bcr/abl	16
**30**	m	AML	58	M	PBSCT	no			8

**31**	f	CML	46	C	PBSCT	no		bcr/abl	14
**32**	m	ALL	42	M	PBSCT	no			11
**33**	m	CML	46	C	PBSCT	no			6
**34**	m	CML	51	M	PBSCT	no		bcr/abl	3
**35**	m	AML	48	M	PBSCT	yes	relapse/+321	trisomy 8	7

**36**	m	CML	43	M	PBSCT	no		bcr/abl	22
**37**	f	CML	38	C	PBSCT	no		bcr/abl	14
**38**	f	AML	34	C	PBSCT	no			21
**39**	f	AML	53	M	PBSCT	no			12
**40**	f	AML	59	M	PBSCT	no			24

**41**	m	CML	50	M	PBSCT	yes	relapse/+62	bcr/abl	3
**42**	f	AML	58	M	PBSCT	no			7
**43**	m	AML	27	C	PBSCT	yes	relapse/+436		7
**44**	m	CML	52	M	PBSCT	no		bcr/abl	9
**45**	f	CML	44	C	PBSCT	no		bcr/abl	9

**46**	m	AML	46	C	PBSCT	no			6
**47**	m	AML	40	C	PBSCT	yes	relapse/+484		7
**48**	m	AML	50	M	PBSCT	no		trisomy 8	17
**49**	f	AML	50	M	PBSCT	no			9
**50**	f	AML	61	M	PBSCT	yes	relapse/+286		5

**51**	m	ALL	66	M	PBSCT	yes	relapse/+701		3
**52**	m	CLL	58	M	PBSCT	yes	infection/+599		4
**53**	m	MM	49	M	PBSCT	no			5
**54**	m	Lym	45	C	PBSCT	yes	relapse/+402		**3**
**55**	m	SAA	35	C	PBSCT	no			3

**56**	m	CML	37	C	PBSCT	no			2
**57**	f	AML	20	C	PBSCT	no			3
**58**	f	ALL	26	C	PBSCT	no			4

m = male, f = female, SAM = severe aplastic anemia, ALL = acute lymphatic leukemia, AML = acute myloid leukemia, CLL = chronic lymphatic leukemia, CML = chronic myeloid leukemia, Lym = lymphoma, MDS = myelodysplastic syndrome, MM = multiple myeloma, NHL = Non-Hodgkin lymphoma, OP = osteopetrosis, n.k. = not known, C = classical, M = metakin, BMT = bone marrow transplantation, PBSCT = peripheral blood stem-cell transplantation.

**Figure 4:**
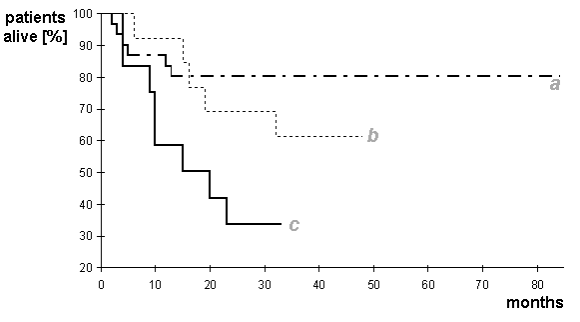
**Survival rates of 33 patients without residual cells (line a), 13 patients with residual cells < 4% (line b) and 12 patients with residual cells > 4% (line c)**.

Patients after dose reduced conditioning treatment prior to transplantation (RC) showed a tendency to develop ≥ 4% residual cells whereas in myeloablative repertoire regimes (MRR) patients trend to develop < 4% residual cells. In detail 54% of patients with ≥ 4% residual cells underwent MRR and 38% RC conditioning. In contrast, 25% of patients with ≥ 4% residual cells underwent MRR and 67% RC conditioning. The survival rate of all three groups (complete chimerism, < 4% residual cells and ≥ 4%) is shown as a Kaplan-Meier-plot in Fig. 4. For a detailed compilation of causes of death see in Table 1.

**Figure 5:**
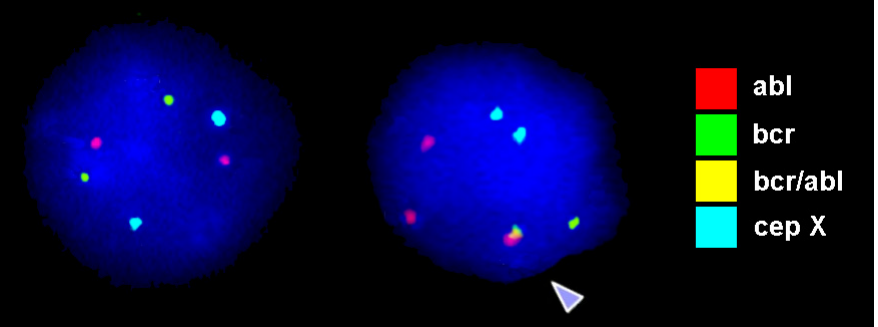
**Bcr/abl negative cell (left) and bcr/abl positive cell (right)**. The right cell shows the bcr/abl-gene-fusion (arrowhead). The LSI ES bcr/abl probe of Vysis/Abbott was applied here.

In 12 patients cytogenetic disease-markers were detected before transplantation (bcr/abl-fusion (n = 9); trisomy 8 (n = 3)). For these cases a simultaneous hybridization of the centromeres X and Y together with the bcr/abl- or centromere 8-probe was performed. As shown above it is possible to decrease the cut off level for acceptor cells by targeting gonosomes and tumour specific genome alterations in a single hybridization. 55 samples of twelve patients were investigated. In nine of these samples residual cells were found in a range of 0.1–3.3%. In two of those nine patients (cases 3, 14) the detected sexmismatch cells were not disease-marker positive, whereas in the other seven patients (cases 29, 31, 35, 36, 37, 41, 45) disease-marker positive residual cells were detected (in total 25 cells). Two specimen of disease-marker negative and disease-marker positive residual cells are shown in Fig. 5.

In these remaining groups with small numbers of patients the disease-marker gave no additional information. The amount of disease-marker-positive or negative residual cells showed no correlation with clinical outcome like relapse or death. Table 2 shows the course of 9 patients with residual cells with known disease-marker.

**Table 2:** Nine patients with disease-marker positive and disease-marker negative residual cells

patient	month										
no.	1	2	3	4	5	6	7	8	9	10	11
4	U^3,0%^				**(2,8%/0%)**						
15	U^1,7%^	U^1,7%^	U^1,7%^	U^1,7%^	U^1,7%^				U^2,3%^		
30					U^2,1%^		**3,5%**		**(1,5%/90%)**	U^0,9%^	U^0,9%^
32	U^2,4%^	U^1,7%^	**(0,2%/100%)**		U^2,1%^	U^2,6%^	U^2,2%^	U^2,2%^	U^2,1%^		U^2,2%^
37	**(0,2%/100%)**	U^1,2%^	U^1,3%^	U^1,4%^	**2,5%**	**39%**		U^1,1%^		+	
38	**(0,4%/25%)**	U^0,9%^	U^0,9%^	U^1,2%^	U^1,1%^	U^0,9%^		U^0,9%^	U^1,2%^		U^0,9%^
39	U^2,7%^				**(0,1%/100%)**				U^1,7%^	**(0,2%/50%)**	U^2,6%^
43	**(0,8%/100%)**	**(6%/0%)**	**64%**	+							
48	U^1,7%^	U^1,7%^	**(0,3%/100%)**	U^1,7%^	U^1,7%^				U^1,7%^		U^2,4%^

patient	month										
no.	12	13	14	15	16	17	18	19	20	21	22

4								+			
15	**(3,3%/0%)**	U^2,2%^	U^1,7%^		U^1,7%^	U^1,7%^		U^1,7%^	U^1,7%^	U^1,7%^	
30	U^1,5%^	U^1,5%^				U^1,5%^	U^1,5%^		U^1,4%^	U^1,5%^	U^1,3%^
32	U^1,7%^		U^2,2%^					U^2,2%^	U^2,2%^		
37											
38	U^1,3%^		U^0,9%^	U^1,3%^	U^1,2%^	U^1,2%^		U^1,3%^	U^1,2%^		
39		U^2,6%^	U^2,5%^			U^2,4%^					U^2,5%^
43											
48	U^1,7%^				U^2,2%^						

patient											
no.	23	24*/25	26	28	29*/30	32	33	35	38*/39	48	

4											
15		U^2,2%^*			U^2,2%^*		U^2,2%^				
30	U^1,3%^	U^0,9%^	U^0,9%^	U^1,3%^		+					
32		U^0,9%^*									
37											
38	U^1,1%^		U^1,1%^		U^1,1%^		U^1,1%^	U^1,3%^	U^1,2%^*		
39				U^2,5%^			U^2,3%^		U^1,7%^	U^2,2%^	
43											
48											

U^X% ^= under cut off level of X%, in brakets are samples were simultaneous hybrization of gonosomes and disease-marker probes was applied, first percentage = amount of residual cells, second percentage = fraction of disease-marker positive residual cells.

## Discussion

### Advantage of automatic scanning system "Metafer"

Chimerism analysis after sex-mismatched bone marrow or peripheral blood stem-cell transplantation is an important diagnostic component to monitor transplantation and minimal residual disease and DLI [15-17]. The FISH technique progresses in importance but demands high personnel skills and costs. An automatic chimerism analysis system could be the solution for that dilemma and was evaluated here. As advantage of automatic analysis using Metafer turned out, that the picture and the coordinates of each cell are memorized. Via basing points it is possible to relocate each cell for further investigation. Moreover, it is possible to analyze huge amounts of cells and to detect small subpopulations of residual cells. Automatic analysis correlates linearly with manual analysis (R^2 ^= 0.985) [18]. This permits to compare automatic and manual chimerism analysis. Because of the small fraction of the targeted cells in the whole population one should analyze in future studies more than 1600 cells for a reasonable precision (rel. standard deviation of 25%) as described in the statistical part.

### FISH vs. PCR

Comparing FISH and PCR in chimerism analysis it was shown that the results are in concordance [7]. The sensitivity of PCR is between 3–5% and allows only semiquantitative analysis [4,19] whereas FISH is more sensitive (1%) and is absolutely quantitative [20]. Because of the different sensitivities it is recommended to use just one method [21], in patients with complete chimerism the method with the highest sensitivity should be used for early detection of residual cells [17,22].

### Evaluation of cut off levels and possible source of error

For cut off levels in XY-FISH-analysis it could be shown that they depend on the sum of evaluated cells per sample (e.g. 3,000 counted cells: cut off 1.2% and 0.6% respectively – see Fig. 3. Trakhtenbrot and coworkers [23] described a very alike cut off level in female cells.

In women with sons, male cells could be found 27 [24] or 38 years [25] postpartum in blood samples. The authors showed that up to 40.000 male cells could be transplanted in a normal PBSCT by female donors. In case male cells were found in patients after transplantation this would be incorrectly interpreted as residual cells. Therefore, listing of female donors with sons is recommended in anamnesis and should be considered in chimerism analysis. Unfortunately, this information was not available for the investigated patients.

In the present study in two samples residual cells were arranged in conglomerates (patient 36, in sample 20 and 26 month after transplantation). The histological origin of these cells was not investigated. Potentially these cells resembled endothelial cells that derived from injury of the endothelium. To prevent contamination with endothelial cells samples from the third aspiration of a single venous puncture is recommended for the cytogenetic analysis. The false positive rate of 1.2% for trisomy 8 we determined was in concordance with Jenkins et al. [26] and Cuneo et al. [27]. The bcr/abl-false positive rate of 0.7% was identical with Amiel et al. [28], Van den Berg et al. [29] and Mühlmann et al. [30]. However, overall a big variation can be found in the literature concering false positive rate of bcr/abl which is given between 2–10% [20,31-34]. Possible reasons for these differences could be: 1) different tissue samples (bone marrow vs. peripheral blood), 2) different cell cycle stage (G1, G2) or 3) different chromatin structure in healthy and moribund cells [35] and 4) different probes. With 95-quantil the cut off level for simultaneous hybridization of gonosomes and disease-markers were estimated and represent 0.005% in XX+trisomy 8/XX+bcr/abl and 0.003% in XY+trisomy 8/XY+bcr/abl. This allows detecting *one *disease-marker positive residual cell in 20.000 analyzed cells which was claimed already in 1994 [36]. PCR as alternative diagnostic method does not have this high sensitivity.

### Automatic scanning applied on sex-mismatched patients

33 patients out of 58 had a complete chimerism, 13 patients residual cells < 4% and 12 patients residual cells > 4%. As expected the detection of residual cells > 4% correlated with relapse as described in literature [37,38]. 66.7% out of patient group > 4% residual cell died because of relapse. Median survival from detection of residual cells and relapse was 6 month and is identical with the data published by Uzunel et al. [39]. Other studies could not find a correlation between mixed chimerism and relapse [40-42]. To what extend mixed chimerism gives evidence about relapse is discussed controversially. In here presented data the occurrence of residual cells was not a marker for relapse. One reason might be the retrospective analysis of patients in this work.

In 12 patients a simultaneous hybridization of gonosomes and disease-markers was applied. 7 patients had disease-marker positive residual cells. But the study showed also that disease-marker positive and disease-marker negative residual cells can be verified within a sample. The detection of disease-marker positive residual cells had no impact on relapse or survival. In contrast Führer et al. [14] could detect disease-marker positive residual cells before relapse. Thiele et al. [13] also arranged a simultaneous hybridization of gonosomes and disease-markers and assumed that cells carrying the disease-marker represent the source for later relapse.

In 3 samples only disease-marker negative residual cells were found. They might represent 1) healthy (benign) leucocytes, 2) precursor tumor cells which do not yet carry the disease-marker, 3) false negative disease-marker positive cells, 4) endothelial cells or 5) cells from female donors with sons.

## Conclusion

Automated chimerism analysis is a robust and sensitive method which can be used in routine diagnosis to detect residual cells effectively and economically. Simultaneous hybridization of gonosomes and disease-marker represent a sensitive method to detect disease-marker positive residual cells with a very low cut off level. The amount of residual cells correlates with survival. There are patients with residual cells < 4% without tendency of relapse. The detection of disease-marker positive residual cells up to 1.3% does not correlate with relapse. Disease-marker positive and disease-marker negative residual cells can appear at the same time in one sample. The definite origin of disease-marker negative residual cells is unclear and should be investigated in a large multicenter study.

## Methods

### Controls

Peripheral blood samples of 21 clinically healthy male (11) and female (10) between 6 and 67 years were studied as controls.

### Patients

A total of 28 female and 30 male patients were analyzed retrospectively after sex-mismatched stem cell transplantation which were performed between 1995 and 2006 at the University Medical Centre Jena. As shown in Table 1, there were 24 acute myloid leukemia (AML), 16 chronic myeloid leukemia (CML), 5 acute lymphatic leukemia (ALL), 4 multiple myeloma (MM), 3 severe aplastic anemia (SAA), 2 myelodysplastic syndrome (MDS), and 1 patient each with Non-Hodgkin lymphoma (NHL), chronic lymphatic leukemia (CLL), lymphoma (Lym) and osteopetrosis (OP). Conditioning regimens were dose reduced in 30 patients or myeloablative in 23 patients [44-46] 54 patients underwent peripheral blood stem-cell transplantation (PBCST) and the remaining 4 bone marrow transplantation (BMT). The median age of the transplanted patients was 46 years (2–89 years). 12 patients showed cytogenetic disease-marker in their malignant cells, i.e. a bcr/abl-fusion in nine and a trisomy 8 in three patients. Overall, 19 patients died, either due to relapse (n = 9), a graft-versus-host-disease (n = 3) or an infection (n = 4). In 3 patients the reason of death remained unclear.

### Cytogenetics and molecular cytogenetics including FISH analysis

Standard techniques were used to cultivate leukocytes out of venous blood, prepare chromosome-preparations [43], and to perform interphase FISH analysis [44]. Commercially available probes (Abbott, Wiesbaden, Germany) for LSI-ES bcr/abl, centromere 8, X and Y were applied.

### Automatic chimerism analysis

For automated analysis we used an Axioplan 2 Imaging microscope (Carl Zeiss Jena, Germany) equiped with CCD-camera CV-M1, 1280 × 1024 pixel (Jai Glostrup, Denmark) and a motorized stage with 8 slide positions (Märzhäuser, Wetzlar, Germany). All components were connected to a personal computer (Dell, Langen, Germany) running the Metafer/MetaCyte-Software from MetaSystems (Altlussheim, Germany).

The evaluation procedure of FISH-slides was as followed: 8 slides were automatically scanned over night and the amount of residual cells was registered. Cells which did not have the characteristic signal combination for XX and XY were excluded. All detected potential residual cells were visually controlled by microscope and each valid cell was further examined wether the residual cell carried a disease-marker or not. The system allowed repositioning of all residual cells in order to visually control the group of interest.

Table 3 shows the parameters used for automated scanning.

**Table 3:** Parameters used for automated scanning

**Parameter/Group**	**Values**			**Description**
**Capturing**				
Color Channels	DAPI	SpO/SpA (X)	FITC/TRITC (Y)	
Max. Integration Time	1.0 s	0.5 s	0.33 s	For capturing images with comparable signal intensities, automatic integration time adjustment was used to reach a certain saturation level in the images while the maximum integration time was limited to 0.5 s (green) and 0.33 s (red) for keeping the background level at low intensities for empty image fields (e.g. not showing signals).
Saturation Area	4 *μ*m^2^	0.7 *μ*m^2^	1 *μ*m^2^	
N Focus Planes	1	5	5	Due to the fact that nuclei are not perfectly flattend on the glass slide by preparation but show Z dimensions within a certain range, the fluorescently labeled chromosomes may be randomly localized in the nucleus also in Z direction.
Distance	0 *μ*m	0.75 *μ*m	0.75 *μ*m	To image the FISH spots perfectly focused, for each signal channel 5 focus planes are captured with a distance of 0.75 *μ*m (this correlates with the depth of field of the objective lens used). These focal planes are then combined to an "Extended Focus Image" which is used for analysis later.
CCD Gain	400%			A CCD camera gain factor was specified to reduce the integration times needed and thereby increase the scanning speed. With the value specified the electronic noise in the captured images was still negligible.
Use CS Mask during Capt	Yes			This parameter was activated to use the counterstain mask for integration time adjustment. As bright artifacts within the image field would usually interfere with the automatic integration time adjustment, using the counterstain mask enabled correct adjustment for image fields where such artifacts were only present outside the nuclei.

**Image Processing**		MedianV	MedianV	An image processing operation was applied to the signal channels to reduce the noise level without significantly reducing the sharpness of the image by vertical median filtering. This filtering was used to remove small "hot spots" of one pixel size in the images which appear in CCD camera images after long integrations or due to camera pixel defects.

**Cell Selection**				
Obj. Threshold	23%			An object threshold of 23% in the counterstain channel was used to segment the cell nuclei. The value is a percentage based on the total contrast range of the captured image.
Min. Nucleus Area	18 *μ*m^2^			The minimum/maximum area in *μ*m^2 ^for a single cell nucleus to be accepted for analysis was used e.g. to exclude (larger) cell clusters.
Max. Nucleus Area	200 *μ*m^2^			
Max. Rel. Conc. Depth	0.4			This criterion has been used to discriminate single cells (showing a convex contour with only small concave areas) from cell clusters (which usually have large concavities). The limit is specified relative to the nucleus diameter.
Max. Aspect Ratio	2.8			This criterion has been used to discriminate the nuclei of interest from more elongated objects. It specifies the maximum ratio of the nucleus diameters along the long and the short principal axis.

**Cell Processing**				
CS/R/G	SBHistoMax ApplyMask			Additional image processing was applied to reduce background/exclude image content outside nucleus contour.
Extend CS Mask	0.5 *μ*m			To correctly identify signals on the nucleus edge the counterstain mask has been extended by 0.5 *μ*m.
**Features/Spot Counting**				
Max. Spot Rel. Area		100/1000	15/1000	To differentiate true FISH spots from variations in the fluorescence background, an upper limit for the relative area of a spot, compared to the whole nucleus (in units of 1/1000) was defined, This was mainly of interest for the green channel (Y chromosome).
R (X)		SpotCounts (5,27)		The number of red FISH spots was determined. Spots were accepted (counted) if they had a minimum distance of 0.5 *μ*m and a minimum intensity of 27% compared to the brightest spot in the same cell.
		Reject if > 2		Cells with more than 2 red spots were automatically rejected.
G (Y)			SpotCounts (14,78)	The number of green FISH spots was determined. Spots were accepted (counted) if they had a minimum distance of 1.4 *μ*m and a minimum intensity of 78% compared to the brightest spot in the same cell.
			Reject if > 2	Cells with more than 2 green spots are automatically rejected.
Reject if No Spots		Yes		Cells not showing any X signals are automatically rejected.

Most important parameters for the classifier used for analyzing the patient samples.

## Competing interests

The authors declare that they have no competing interests.

## Authors' contributions

JE carried out the cytogenetic work, was involved in performing the statistical analysis and conceived the manuscript. IH and AH made substantial conclusions and performed initial tests. HS performed initial tests. MH performed the main part of the statistical analysis with help of TK and AG. HGS made substantial contributions to acquisition of data and gave final approval of the version to be published. TJ extracted the software parameters for Metafer. UC made substantial conclusions to conception and design. TL drafted the manuscipt. IFL designed the structure and coordinated the study. All authors read and approved the final manuscript.
